# Rogue waves in disordered 1D photonic lattices

**DOI:** 10.1038/s41598-020-69826-x

**Published:** 2020-08-03

**Authors:** Danilo Rivas, Alexander Szameit, Rodrigo A. Vicencio

**Affiliations:** 1 Departamento de Física and MIRO, Facultad de Ciencias Físicas y Matemáticas, Universidad de Chile, Santiago, Chile; 2 Institute for Physics, University of Rostock, Albert-Einstein-Strasse 23, 18059 Rostock, Germany

**Keywords:** Optics and photonics, Photonic crystals, Electronics, photonics and device physics, Photonic devices, Complex networks

## Abstract

In this work, we study the phenomena of Rogue waves (RW) on one-dimensional (1D) photonic lattices presenting diagonal and non-diagonal disorder. Our results show the appearance of extreme events coming from the superposition of different, extended and localized, linear waves for weak disorder. We perform experiments on femtosecond laser written waveguide arrays having disorder in coupling constants, which is originated from a random waveguide distribution. Both, numerics and experiments, are in good agreement and show that RW are generically present in 1D lattices for weak disorder only, after a mandatory data filtering process.

## Introduction

Rogue waves (RWs) are old phenomena, probably emerging at the very beginning of the universe. However, they were first described as large amplitude water waves on open ocean, suddenly appearing and disappearing without any cause and, sometimes, producing serious damages on ships^1,2^. These waves are classified as extreme events (EE) in the sense of statistic due to their rare appearance but high amplitude, which is associated with long tails distributions. The origin of such extreme wave phenomena has been controversial, dividing the approaches between linear and nonlinear wave mixing processes^3^. For example, wave tanks could naturally induce nonlinear phenomena due to their confining, which is completely absent in open sea, where waves suffer the interaction with other waves. Interestingly, due to the changes in global weather, an important increment in occurrence and severity of RWs have been reported^4^, making even more important their study due to the emergent damage on populated areas.

Although initially RWs were applied to description of ocean phenomena, nowadays they are an important subject of complex systems research, going from oceanography, optics, and biology, to sociology, economy, etc.^5^. Particularly in optics, nonlinear Schrödinger-like models^6–8^ have associated the appearance of EEs with the excitation of coherent structures, including modulational instability and self focusing and defocusing processes^9–12^. Recently, analogies between light and ocean phenomena have been reported^13^, including results supporting linear and nonlinear interpretations.

Discrete systems^14,15^ have been a bit outside of this discussion and only few numerical results have been reported to date^16–18^, which are focused mostly on nonlinear lattices presenting disorder. In particular, in Ref.^18^ authors numerically found that weak disorder has an important effect on the appearance of Rogue Waves, results that could be associated with the concept of caustic effects coming from purely linear, large-amplitude events on an optical sea^19^. In discrete linear lattices, dynamics is governed by ballistic propagation, having a characteristic Discrete Diffraction (DD) pattern when exciting a single bulk site^14^. This implies that energy spreading across the lattice is mediated by the excitation of linear propagating waves, which explore the system with different velocities depending on their specific *k*-vector. On the other hand, disordered lattices have shown to produce Anderson-like localization^20–22^ due to a continuous destructive interference of these propagating waves on a disordered landscape. This produces large amplitude and highly localized profiles through the lattice. However, this is an inevitable property which, therefore, can not be considered as a rare or extreme event.

In this work, we focus on searching for RWs on discrete disordered photonic lattices. In particular, we study a one-dimensional system by performing numerical simulations considering diagonal (on-site) and off-diagonal (coupling) disorder. We found that weak disorder becomes an ideal regime for observing extreme events. There, wave transport and weak trapping effects are simultaneously possible, which naturally help to facilitate constructive interference, with the possibility to excite large amplitude waves, as causticity suggests^12,19^. In addition, to our knowledge, we report on the first experiments on femtosecond written photonic lattices^23^ looking for this phenomena. We corroborate the numerical findings and validate the application of an intensity filter as an important step in the data analysis, in order to distinguish between purely extreme and spurious events.

**Figure 1:**
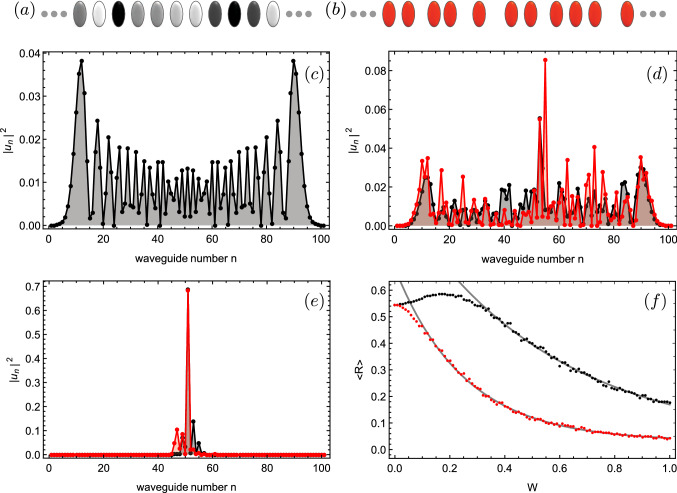
(**a**, **b**) diagram for lattices presenting diagonal and non-diagonal disorder, respectively. Intensity output profile at $z=z_{max}$ for $w_\beta (w_V):$ (**c**) $0\ (0)$, (**d**) $0.3\ (0.15)$ and (**e**) $1\ (1)$. (**f**) Averaged output participation ratio *R* versus $W_{\beta }(W_V)$ for 100 realizations (gray lines correspond to an exponential fit). Black (red) color is for diagonal (non-diagonal) disorder. $N=101$ sites, $V_0=1$, and $z_{max}=21$.

## Model and numerical results

We study the propagation of light in one-dimensional photonic lattices, including on-site and coupling disorder. By using a coupled-mode approach, assuming the excitation of fundamental waveguide modes only, we obtain a set of normalized discrete linear Schrödinger equations^14,15^ written as

1 $$\begin{aligned} -i\frac{\partial u_n}{\partial z} =\beta _n u_n + \sum \limits_{m \ne n} V_{n,m} u_m .\end{aligned}$$

Here, $u_n$ describes the electric field amplitude at the *n*-th site of a lattice having *N* waveguides, and *z* corresponds to the propagation coordinate or dynamical variable. Function $\beta _n$ defines the distribution of on-site disorder across the lattice. Experimentally speaking, this term is related to different propagation constants which can be implemented by fabricating waveguides with different refractive index contrasts and/or different waveguide geometries^23^, as sketched in Fig. 1a. $\beta _n=\beta _0$ defines a system formed by identical waveguides only. Term $V_{n,m}$ defines the coupling interaction between nearest-neighbor *n* and *m* waveguides. This coefficient depends on waveguide parameters but, more precisely, it decays exponentially with distance^23^. So, experimentally speaking non-diagonal disorder is implemented by randomly choosing the distance between waveguides on a given lattice which is, in fact, the experimental case we will consider in this work [see Fig. 1b].

We are interesting on studying the effect of isolated disorder, diagonal or non-diagonal, in order to identify the appearance of Rogue waves in each case. First of all, we start by studying the dynamics of a lattice with on-site disorder only. In this case, we define $\beta _n=\beta _0 + \Delta \beta$ with $\Delta \beta \in \{-W_\beta ,W_\beta \}$, and $W_\beta \in \{0,2\}$ the strength of disorder. Without loss of generality, we set $\beta _0=0$, which does not affect the physics of the problem, but a shift in propagation constants. In this case, we set function $V_{n,m}=V_0$, with all waveguides separated by the same distance, as described in Fig. 1a. Considering this, model (1) becomes

2 $$\begin{aligned} -i\frac{\partial u_n}{\partial z} =\Delta \beta u_n+V_0(u_{n+1}+u_{n-1}). \end{aligned}$$

In general, as we use $V_0=1$ in numerics, the disorder strength is always implicitly normalized to $V_0$. First of all, we study the global dynamics of model (2) by initializing the system with a single-site excitation: $u_n(0)=\delta _{n,n_c}$, with $n_c$ the lattice center. Figure 1c–e show (black connected dots) typical profiles at different disorder levels. For zero disorder, we observe a discrete diffraction pattern that disseminate the energy across the lattice with two typical main propagating lobes. While increasing the disorder strength, the output profile is composed of a discrete diffraction profile plus randomly localized peaks, depending on the particular disorder distribution. This naturally increases the efficiency for energy spreading due to a better densification of the excited area. When disorder strength is high enough, the energy gets trapped due to Anderson localization, which is originated by destructive interference of incoming and outcoming waves^20^. We use the participation ratio *R*, defined as $R=(\sum _n |u_n|^2)^2/(N\sum _n |u_n|^4)$, as an energy distribution indicator. This quantity measures the number of relevant excited peaks on a given spatial profile: a single peak on a lattice gives $R=1/N$, while a lattice equally excited gives $R=1$ (profiles with $R\gtrsim 0.4$ are considered as delocalized^24^). As it was already shown, theoretically^24^ and experimentally^25^, for diagonal disorder, the distribution of energy increases for an increasing level of disorder, achieves a maximum and, then, decreases for stronger disorder. In Fig. 1f we present our results after averaging the participation ratio at the output position $z=z_{max}$ versus the disorder strength $W_{\beta }$ (black dots). We also include an exponential fit to separate regimes of energy spreading and Anderson-like localization (where we expect an exponential decaying tendency). As we will describe below, the region where $R\gtrsim 0.5$ for $W_{\beta }\lesssim 0.3$ corresponds to a favorable dynamical regime for observing wave interaction and, as a consequence, an increasing EE count.

A second study corresponds to consider a lattice presenting off-diagonal disorder only, which is implemented by taking random distances between waveguides [see sketch in Fig. 1b] and keeping the propagation constants fixed for all of them (without loosing generality, we simply set $\beta _0=0$). In this case, we write the disorder as $V_{n,m}=V_{m,n}=V_0(1+ \Delta V)$ with $\Delta V \in \{-W_V,W_V\}$, and $W_V \in \{0,1\}$. Here, the disorder strength can not be larger than 1 in order to avoid negative coupling constants, which are absent in model (1), that considers the excitation of fundamental modes only^26–28^. Therefore, the model for off-diagonal disorder simply reads as

3 $$\begin{aligned} -i\frac{\partial u_n}{\partial z} =V_{n,n+1}u_{n+1}+V_{n,n-1}u_{n-1}\, \end{aligned}$$

where equations for amplitudes $u_{n\pm 1}$ consider the coupling condition $V_{n,n\pm 1}=V_{n\pm 1,n}$, which is necessary in order to correctly define a realistic disordered lattice and to fullfill the Power and Hamiltonian conservation of model (1). Figure 1c–e show some examples at different disorder levels. The main differences are that comparable on-site regimes occur at different strength of disorder, as expected considering that the off-diagonal case somehow reduces the effective propagation area, when coupling becomes too small on a given direction. A weak coupling reduces the chances for light to propagate through that direction and, therefore, the effect of non-diagonal disorder on dynamics is stronger (more aggressive). The tendency of averaged output participation ratio versus disorder strength [red dots in Fig. 1f] is different compared to the diagonal case for weak disorder. However, as the exponential fit shows, the region where $R\gtrsim 0.45$ and away from an exponential decaying tendency occurs for $W_{V}\lesssim 0.1$. Again, this region corresponds to a favorable regime for wave interaction, where we expect to count a larger amount of EEs.

## Rogue wave analysis

Formally speaking, a Rogue Wave has been defined, in the oceanographical context, as an extreme wave appearing suddenly and having an amplitude larger than the rest of a given amplitude ensemble. This gives a very low probability for the excitation of such an extremely large wave, been therefore a rather rare event. Accordingly, it becomes necessary to give a technical definition in order to study this phenomena on different contexts. In this way, we require to compute a given quantity that could be compared for different system parameters in order to clearly define if the large amplitude events found in the dynamics are extreme or not. Along this work, we will define that an intensity $I_n\equiv |u_n|^2$ corresponds to an extreme event if $I_n>2I_s$, where $I_s$ corresponds to a threshold intensity. $I_s$ is defined as the average value of the highest intensity tertile of the corresponding probability density function (PDF) distribution^12^. Therefore, for a given realization, we analyze all sites intensities $I_n$ and obtain a PDF distribution where we define the intensity $I_s$ and count the number of EEs over the entire intensity ensemble.

**Figure 2:**
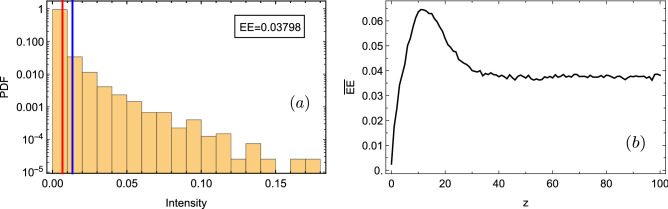
(**a**) Semi-log PDF distribution for a single realization of diagonal disorder ($W_{\beta }=0.1$), and for a given propagation distance $z=95$. Red and blue vertical lines indicate $I_s$ and $2I_s$, respectively. (b) Percentage of EEs versus propagation distance *z* for same realization described in (a).

As the intensity distribution and, therefore, the number of EEs change at different propagation distances, we develop a full dynamical characterization: For a given realization of disorder, we numerically integrate model (1) in the interval $z\in \{0,z_{max}\}$. Then, for every step in *z*, we analyze the corresponding spatial profile $\ |u_n(z)|^2$ and count the lattice intensities to create a PDF distribution, where we identify the percentage of EEs. Figure 2a shows an example of a PDF distribution for diagonal disorder and for a given distance *z*. We observe a heavy-tailed intensity distribution, what is an indicator of the existence of EE^29^. For every step in *z*, we obtain the percentage of EEs versus propagation distance, as shown in Fig. 2b. Here, we observe an increasing number of EEs versus distance, which is associated with light spreading across the lattice and reflections occurring for $z\gtrsim 10$. As a reflected wave superposes to slower propagating fronts, this causes a spurious increment of local intensities $I_n$, what adds extra counts to the overall statistic. Afterwards, light spreading is more homogeneous and the number of EEs reduces up to a rather constant value ($\sim 0.38$ in the example), observing some kind of thermalization or saturation phenomena. This means that the spatial pattern, although still fluctuating, behaves similarly for an increasing value of z. We, therefore, observe an EE saturation after some propagation distance, what strongly depends on the degree of disorder.

**Figure 3:**
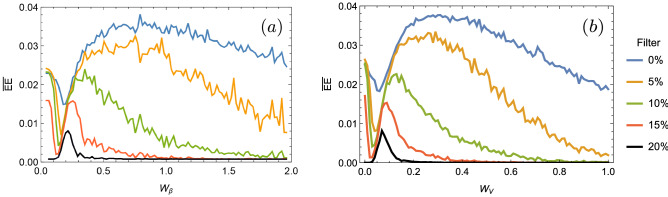
Averaged extreme events ${\overline{EE}}$ versus disorder strength for (**a**) diagonal and (**b**) non-diagonal disorder. Different colors corresponds to different applied filters, as indicated in figure.

Taking this information into account, we define an EE dynamically averaged value (${\overline{EE}}$), obtained by averaging the number of EEs in the interval $z\in \{85,95\}$ for a given realization and given disorder, and then averaging again over 100 disorder realizations. In the chosen *z*-interval, dynamics has already relaxed, without the strong effect of first reflections at surfaces. So, we can correctly characterize the appearance of large amplitude events for a given degree of disorder. We collect the information of ${\overline{EE}}$ versus disorder strength in Fig. 3a, b, for diagonal and non-diagonal disorder, respectively. Blue curves show the averaged data as described above, which consider all lattice amplitudes, without any filter, when counting intensity peaks to form the corresponding PDF distribution. We observe a rather strange behavior in both blue curves. First, ${\overline{EE}}$ decreases to a minimum around $\{W_{\beta },W_{V}\}\sim \{0.15,0.05\}$, from zero to weak disorder, and then ${\overline{EE}}$ increases to a maximum located close to $\{W_{\beta },W_{V}\}\sim \{0.6,0.3\}$. Afterwards, in both cases, the number of ${\overline{EE}}$ decays slowly. We observe that the typical discrete diffraction pattern, having two characteristic main lobes [as shown in Fig. 1c], increases the statistic of large amplitude events. Without filtering, all intensity are counted and the intensities at lobes (that include several sites) are been considered as EEs. Of course, this is out of the definition of a low statistic large amplitude and rare event, which is necessary to define a RW. In other words, every time we would propagate light on a homogenous lattice we would observe a RW. Clearly, a RW is a rare event, therefore the described observation (discrete diffraction) can not be categorized as such. This also affects the dynamics at weak disorder, because in that regime propagation is a mixture between discrete diffraction and trapping at disordered regions, as shown in Fig. 1d. On the other side, we know that for larger disorder we will end up with Anderson localization^20^ [see Fig. 1e]. Obviously, this regime can not be counted as a RW, because, as Anderson taught us, this observation will always occur for a disordered system; therefore, it would not be a rare event at all. For a large disorder strength, profiles are completely localized with $R\rightarrow 1/N$, having a single excited site. Therefore, if not filter is included in data, the number of EE must go to the limit of 1/*N*, with only one site having a large amplitude. By filtering the data, this peak will be exactly $I_s$ and, therefore, no RWs will be defined, as it should be. Clearly at this regime, the overall statistic increases when counting all amplitude sites and the localized profile generates spurious large amplitude events.

As our work is focused on identifying the effect of disorder on the appearance of RWs on 1D lattices, we implement a *filtering process*. We first notice that low amplitude peaks increase the amount of data and, therefore, decrease the threshold value $2I_s$. So, we decide to simply avoid counting low amplitude data by applying an intensity filter, which is defined as a given percentage of the largest peak ($I_{max}$) at a given realization. To wit, $X\%$ means that we will only count sites with intensities larger than $X\cdot I_{max}/100$. The result of applying this filtering process is presented in Fig. 3, using different colors depending on the indicated filter value. First of all, for both cases, we notice that the inclusion of a data filter produces a similar tendency. We observe how the number of averaged ${\overline{EE}}$ decreases for an ordered system, what indicates a correct effect of our suggested filtering process. The previously described minimum decreases and is shifted to a smaller value of disorder for an increasing applied filter. In both cases, we observe that a filter of around $20\%$ completely eliminates the ${\overline{EE}}$ count at zero disorder. So, with this level of filtering we are indeed avoiding to count a non rare effect as discrete diffraction. In addition, our filter is also eliminating Anderson localization as a RW, reducing to zero the averaged ${\overline{EE}}$ for larger disorder (in this regime, a lower filter of $\sim 15\%$ is, in fact, enough). Our filter is indeed shifting the threshold intensity $I_s$ allowing to classify only very rare large amplitude events as RWs. Nicely, considering the dynamics at weak disorder, we have been able to limit the ${\overline{EE}}$ region as a result of the propagation of linear extended waves plus weak random localization on distorted regions across the lattice. That means that, on a 1D disordered photonic lattice, we would observe an extreme event only when a good balance between transport and weak localization effects is achieved and large events become statistically possible. As we observe in Fig. 3, the maximum number of ${\overline{EE}}$, after filtering $20\%$ of peaks, is quite low, with a value lower than $1\%$ of total counted peaks.

## Experiments for off-diagonal disordered lattices

**Figure 4:**
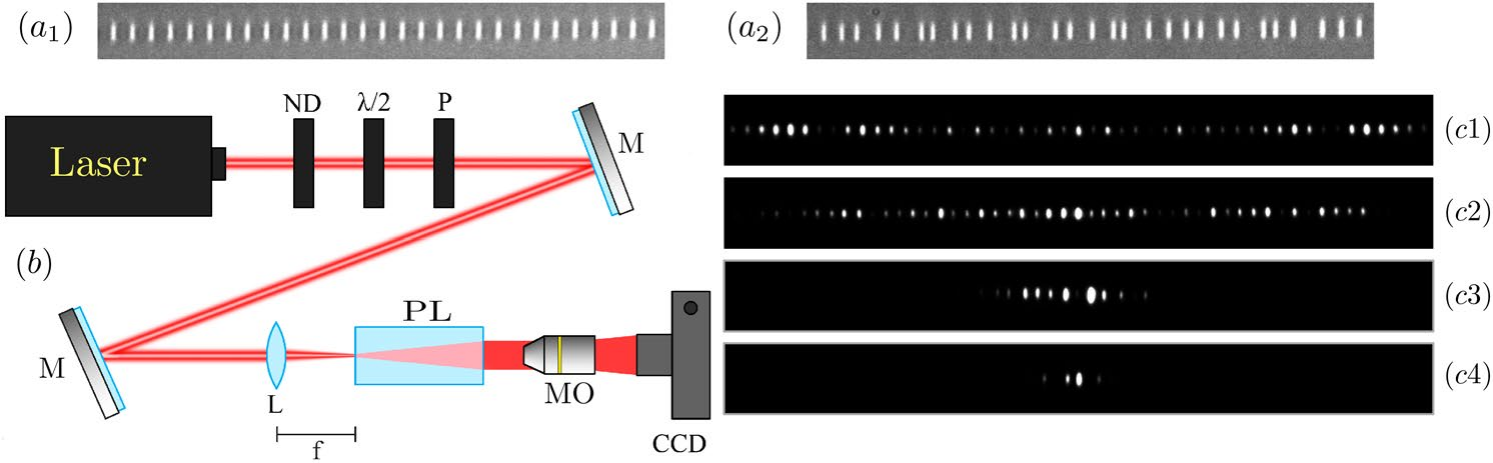
(**a1**, **a2**) Output microscope images for broad white light illumination of an ordered and a disordered waveguide array, respectively. (**b**) Simplified experimental setup: ND is a neutral density filter, $\lambda /2$ a half-wave plate, P a linear polarizer, M a mirror, L a lens, PL a photonic lattice, MO a microscope objective, and CCD a digital camera. (**c1**–**c4**) Output intensity images for single-site laser excitation at $\delta : 0,1,3$ and 6, respectively.

We perform experiments on 8 photonic lattices which were fabricated in fused silica, by femtosecond laser writing technique^23^, to characterize the dynamics on disordered 1D systems^25,30,31^. Each lattice possesses 81 sites, a nominal waveguide separation of $16\ \mu$m, and a propagation distance of 10 cm. Disorder is included by randomly varying the distance between waveguides from the set $\{16-\delta ,16+\delta \}$, with $\delta \in \{0,1,2,3,4,5,6,7\} \, \mu$m. Therefore, $\delta$ is related to the disorder strength $W_V$, and a larger value implies a larger waveguide separation, meaning a weaker coupling interaction, which decays exponentially with distance^23^. Figure 4a1, a2 show microscope images for homogenous ($\delta =0$) and disordered ($\delta =5$) lattices, respectively, after white light illumination.

**Figure 5:**
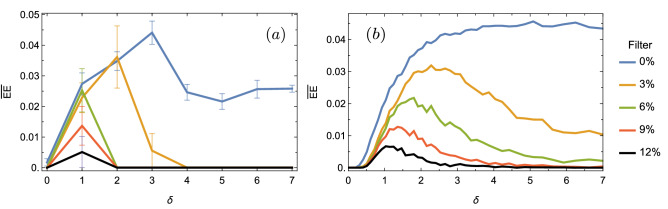
(**a**) Experimentally and (**b**) numerically averaged percentage of ${\overline{EE}}_{exp}$ versus parameter $\delta$. In (**a**) bars indicate the standard deviation for all data. Numerics in (**b**) were performed for $N=81$ sites, $V_0=1.2 \, {\hbox {cm}}^{-1}$, and $z_{max}=10$ cm. Different colors indicate different data filters as described in figure.

The experiment consists on illuminating a single waveguide of a given array, by focusing a 633 nm HeNe laser beam, after power controlling, polarizing, and aligning the beam (see Fig. 4b). Polarization is set to a horizontal one in order to have a larger effective coupling and, therefore, a larger spreading area^31^. This aspect is crucial to observe RWs on photonic lattices, because the interaction between different propagating and localized waves is the main mechanism for an abrupt increment of a given site intensity. As our numerical filtered results suggest, this can be observed for weak disorder, where these mixed regimes can be found. To excite a single site on a given lattice, we use a short focal lens of $f=25.4$ mm and shift the input position transversally using micro-mechanical stages. When injecting light on a single waveguide we are indeed exciting all linear modes which have an amplitude different to zero at that site. Therefore, we expect to excite localized as well as propagating linear waves. We image the output profile on a CCD camera, by using a $10\times$ microscope objective (MO). For zero disorder, we observe discrete diffraction as shown in Fig. 4c1. Then, if we slowly increase the disorder strength, for example to $\delta =1$ (see Fig. 4c2), we still observe a good diffraction pattern but having some localized peaks. This is a good experimental example where we expect to observe an EEs enhancement. Then, by further increasing the disorder strength to $\delta =3$ and larger, we observe an already quite localized profile, as shown in Fig. 4c3 and c4. Figures 4c1–c4 correspond to specific examples at different $\delta$-values. However, in order to accumulate statistic and experimentally determine the appearance of RWs in 1D photonic lattices, we need to measure several images at every photonic lattice. Experimentally speaking, disorder has been historically studied by using a single disordered lattice, but exciting different bulk sites^25,30,31^. This is a valid and more efficient approach to study disorder effects because different lattice positions imply different excited modes and, at the end, a different observed dynamics. In our case, we excited 20 different bulk sites in each of our 8 lattices. With that, we obtained 20 realizations for each degree of disorder and compute the percentage of EEs in a similar way than for numerical data. We collect the information in Fig. 5a. In the experimental case, we only obtain information at the output facet of the lattice at $z=10$ cm. Therefore, we average over 20 output images for every degree of disorder and define ${\overline{EE}}_{exp}$.

In general, we observe a rather similar tendency on the experimental EE count, when comparing with numerical data from Fig. 3b. However, for weak disorder we do not observe discrete diffraction as an EE, what is different compared to numerical findings. When collecting experimental data, the exposition time from the CCD camera is set such that main peaks are not saturated in a scale from 0 to 256, which is a typical scale for digital images [notice that Fig. 4c1–c4 are saturated for presentation purposes]. Therefore, we are always experimentally applying some kind of filtering to the obtained images. This means that low intensity peaks will simply not appear in our counting process because they are in the order of the image background. However, even more important is the propagation distance in experiments. Our simulations in Fig. 3 were obtained for larger propagation distances, because we wanted to describe a more asymptotic regime for 1D disordered lattices, in order to get a more general evidence of EEs in this kind of lattice systems. We also ran numerical simulations for shorter propagation distances as shown in Fig. 5b, in order to compare directly with experiments (we set $V_0=1.2 \, {\hbox {cm}}^{-1}$, where we find good agreement). There, we corroborate that EEs are not appearing at zero disorder. This is quite reasonable because at shorter distances, light spreading occupies a reduced area and the spatial profile is more homogenous and flatter. This naturally implies no counting for EEs at zero disorder because a flatter pattern gives a value for 2*Is* around the value of the pattern and, therefore, no EE is counted.

When applying filtering on experimental data (see Fig. 5a), we observe a stronger effect than in numerics (see Fig. 5b), probably because the overall amount of excited peaks is smaller and statistic lower. So, a data filter has a stronger effect on experiments. Nevertheless, we nicely observe how the highly localized patterns obtained for a large degree of disorder are eliminated as RWs when filtering both, experimental and numerical, data. For a filter of the order of $5\%$ we already eliminate Anderson localization as an EEs and observe the appearance of an EEs peak similar to the one found in Fig. 3. Naturally, when increasing the amount of filtering this peak decreases as the amount of statistical data decreases as well. This is quite evident looking the last experimental used filter of $12\%$, where the standard deviation shows that samples could sometimes show and sometimes do not show large amplitude and rare events.

## Conclusions

In conclusion, we have studied the existence of RWs in 1D photonic lattices having on-site or coupling disorder. In general, we numerically determine the existence of a well defined peak for extreme events at weak disorder. This peak occurs in a region where propagating waves are interfering with weakly localized patterns and, therefore, linearly superposing and generating regions of constructive and destructive interference. This resembles the results found in linear optical systems^19^, where caustic-like effects are the responsible for large amplitude and rare events. We performed a first experiment on 1D disordered photonic lattices and found a similar tendency for averaged data, with the corresponding EE peak at weak disorder. We found a good phenomenological agreement between numerics and experiments and, therefore, validate the appearance of EEs on purely linear disordered systems. One of the main contributions of our work is the definition of a data filter to avoid counting spurious data as a RW. We showed that this filter correctly eliminates always observed phenomena of discrete diffraction and Anderson localization from the general count. We believe that our results could stimulate further experimental investigation of extreme phenomena in photonic lattices, considering different geometries and dimensions, as well as different nonlinearities.

## Methods

### Sample fabrication

The photonic lattice used in our experiment was fabricated using the femtosecond laser writing technique^23^. By focusing a laser beam on a fused silica plate, we are able to locally modify the refractive index. Then, we translate the sample at fixed velocity and create a complete waveguide inside the glass plate. Depending on the transversal pattern of the specific lattice, we repeat this procedure on several positions and fabricate a full photonic system.

## Data Availability

The datasets generated during and/or analyzed during the current study are available from the corresponding author on reasonable request.
